# Study on the Hydrogen Effect and Interface/Border Traps of a Depletion-Mode AlGaN/GaN High-Electron-Mobility Transistor with a SiN_x_ Gate Dielectric at Different Temperatures

**DOI:** 10.3390/mi15020171

**Published:** 2024-01-24

**Authors:** Dongsheng Zhao, Liang He, Lijuan Wu, Qingzhong Xiao, Chang Liu, Yuan Chen, Zhiyuan He, Deqiang Yang, Mingen Lv, Zijun Cheng

**Affiliations:** 1 School of Physics & Electronic Science, Changsha University of Science and Technology, Changsha 410114, China; 22111021977@stu.csust.edu.cn (D.Z.);; 2 The Key Laboratory, The Fifth Electronics Research Institute of the Ministry of Industry and Information Technology, Guangzhou 510610, China; xiaoqz@ceprei.com (Q.X.); xd_liuchang@163.com (C.L.); chenyuan@ceprei.com (Y.C.); hezhiyuan1988@126.com (Z.H.);

**Keywords:** SiN_x_, AlGaN/GaN HEMT, hydrogen, interface trap, border trap

## Abstract

In this study, the electrical characteristics of depletion-mode AlGaN/GaN high-electron-mobility transistors (HEMTs) with a SiN_x_ gate dielectric were tested under hydrogen exposure conditions. The experimental results are as follows: (1) After hydrogen treatment at room temperature, the threshold voltage *V*_TH_ of the original device was positively shifted from −16.98 V to −11.53 V, and the positive bias of threshold was 5.45 V. When the *V*_DS_ was swept from 0 to 1 V with *V*_GS_ of 0 V, the *I*_DS_ was reduced by 25% from 9.45 A to 7.08 A. (2) Another group of original devices with identical electrical performance, after the same duration of hydrogen treatment at 100 °C, exhibited a reverse shift in threshold voltage with a negative threshold shift of −0.91 V. The output characteristics were enhanced, and the saturation leakage current was increased. (3) The C-V method and the low-frequency noise method were used to investigate the effect of hydrogen effect on the device interface trap and border trap, respectively. It was found that high-temperature hydrogen conditions can passivate the interface/border traps of SiN_x_/AlGaN, reducing the density of interface/border traps and mitigating the trap capture effect. However, in the room-temperature hydrogen experiment, the concentration of interface/border traps increased. The research findings in this paper provide valuable references for the design and application of depletion-mode AlGaN/GaN HEMT devices.

## 1. Introduction

The third-generation semiconductor material gallium nitride (GaN) has advantages such as a wide bandgap, high electron mobility, high thermal conductivity, and high saturation electron drift velocity, making it superior to Si and GaAs in terms of radiation resistance and breakdown resistance [1]. In theory, the wide bandgap characteristics make GaN devices highly reliable at high temperatures, meeting the requirements for applications in defense, aerospace, and aviation. However, previous studies on GaAs devices have shown that a small amount of hydrogen (only 5% of atmospheric content) can lead to a decrease in carrier concentration in the device channel, thereby reducing the transconductance and RF performance of the device [2]. In special space environments such as aerospace satellites, GaN devices need to be sealed and packaged [3,4]. However, during the preparation process of the devices, unavoidable H-containing compounds are generated, and the slow release of hydrogen ions trapped in the sealed package leads to the devices being in a hydrogen-rich environment, ultimately affecting their stability and reliability [5,6,7,8]. Therefore, conducting research on the reliability of hydrogen effects in commercial GaN devices for special applications such as deep space exploration is very important and valuable [9,10].

Theoretically, the drift of *V*_TH_ may arise from border traps inside the bulk SiN_x_, traps at the SiN_x_/AlGaN interface, and trap states in the AlGaN/GaN interface and AlGaN barrier. On the one hand, devices with in situ SiN_x_ have better quality and interfaces; On the other hand, the trap density at the amorphous SiN_x_/AlGaN interface is much higher than the density at the AlGaN/GaN interface; that is, the defects at the AlGaN/GaN interface do not significantly contribute to the *V*_TH_ drift [11,12,13]. We therefore propose a guess: the border trap inside SiN_x_ and the interface trap of SiN_x_/AlGaN have a significant impact on the threshold voltage. However, by reviewing the relevant literature, it was found that due to the lack of corresponding characterization techniques, it is difficult to distinguish the influence of amorphous SiN_x_/AlGaN interface trap and border trap on the experiment [5,6,14]. The previous experimental research only describes the interaction between electrons and general interface states, without considering the influence of border traps in gate media [5,6,11]. On this basis, the content of this article mainly focuses on the reliability of hydrogen effects in D-mode GaN HEMT devices at different experimental temperatures. From the perspective of characterization methods, current-based deep-level transient spectroscopy (DLTS) has the advantages of being sensitive to measurement, having a wide range of detectable defect energy levels, being able to simultaneously measure both majority and minority carrier traps, and being able to determine trap positions. But in experiments, DLTS is more widely used to study deep traps, such as bulk traps, while other methods such as C-V are mainly used to characterize the traps at semiconductor interfaces. On the basis of the above, the combination of the C-V method and low-frequency noise method was used to characterize interface traps and border traps in this experiment [15,16,17,18,19], and the mechanism of their electrical characteristic changes was analyzed.

## 2. Materials and Methods

**Figure 1:**
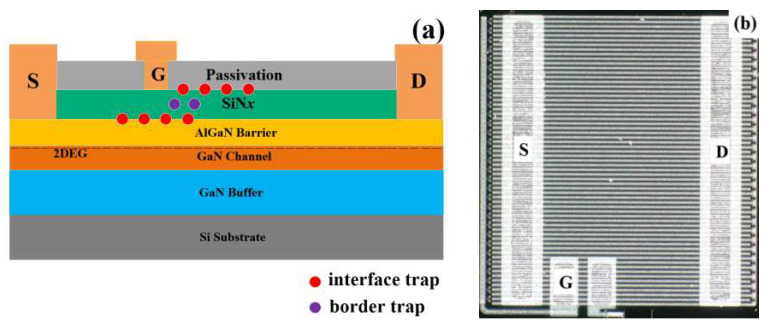
(**a**) The longitudinal cross-section diagram of the depletion-mode AlGaN/GaN HEMT with a SiN_x_ gate dielectric, as well as the positions of border traps and interface traps; (**b**) schematic diagram showing the positions of the chip surface, drain, gate, and source.

Figure 1a shows the longitudinal section of this commercial device. The growth of AlGaN/GaN heterojunction epitaxial layer was carried out via metal-organic chemical vapor deposition (MOCVD) on silicon substrate. Carbon doping was carried out with the GaN buffer layer to acquire high breakdown voltage. The AlGaN/GaN heterostructure quality was improved by depositing an unintentionally doped GaN material as a channel layer to acquire high conduction. In the process of device fabrication, a layer of SiN_x_ was grown through low-pressure chemical vapor deposition (LPCVD) as a gate dielectric layer and passivation layer. The drain/source and gate electrodes were formed by depositing gold-free metals to form ohmic contacts and MIS-gate contact, respectively. In order to avoid the influence of other unrelated factors, six original devices were selected and stored at room temperature at 25 °C for 2 weeks, and then they were divided into group I and group II for the experiment. Experiment I: The device was placed in a hydrogen-rich environment at room temperature of 25 °C, and the data were collected at 200 h and 500 h, respectively. Finally, the device was incubated at 100 °C for another 200 h to evaluate the effect of temperature on the device. Experiment II: The device was placed in a hydrogen-rich environment at 100 °C, and the data were collected at 200 h. The device used to collect DC characteristic data is the Agilent B1500A semiconductor device analyzer produced by Keysight Technologies (Santa Rosa, CA, USA). Low-frequency noise data were obtained through sampling and testing of the low-frequency module of the PRIMARIUS FS Pro (Primarius-Tech, Shanghai, China) low-frequency noise testing equipment. The high-temperature experimental equipment used is a high-temperature constant temperature test chamber produced by the Fifth Electronic Research Institute of the Ministry of Industry and Information Technology (Guangzhou, China).

## 3. Results and Discussion

### 3.1. Impact of Hydrogen Effect on Device Electrical Characteristics

**Figure 2:**
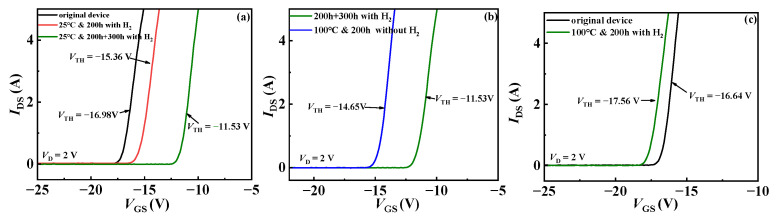
(**a**) Transfer characteristics with hydrogen at 25 °C in experiment I; (**b**) transfer characteristics without hydrogen at 100 °C in experiment I; (**c**) transfer characteristics with hydrogen treatment at 100 °C in experiment II.

As shown in Figure 2a,b, experiment I shows the change in transfer characteristics of hydrogen treatment at room temperature. The threshold voltage *V*_TH_ of the device showed a positive drift, from −16.98 V to −15.36 V, with a drift of 1.62 V. After an additional 300 h of exposure to hydrogen gas, the threshold voltage of the device continued to drift positively, and the amplitude increased. The threshold voltage moved from the previous −15.36 V to −11.53 V, with a drift of 3.83 V. Subsequently, the device was placed in a high-temperature environment of 100 °C to observe the effect of temperature on the hydrogen treatment device. As shown in Figure 2b, after high-temperature treatment, the threshold voltage of the device showed a reverse drift, with a drift amount of −3.12 V. The reason for not being able to recover to the initial state may be the insufficient high-temperature treatment time. In experiment II, the threshold voltage of the device also showed negative drift, moving from the initial −16.65 V to −17.56 V, with a reverse bias of −0.91 V, as shown in Figure 2c. In experiment I, the threshold voltage shifted forward because H_2_ is differentiated into H and H+ states when it encounters the gate metal due to the adsorption. Impurities in the SiN_x_ dielectric layer below the gate can accept electrons to form negative centers and become receptor traps on the SiN_x_/AlGaN interface/border. The threshold voltage drifts to the positive direction due to a large number of receptor traps. Not only the interface trap in the SiN_x_/AlGaN interface but also the deep level trap in the SiN_x_ dielectric layer are passivated by the donor H+ due to the high activity of H+ in experiment II.

**Figure 3:**
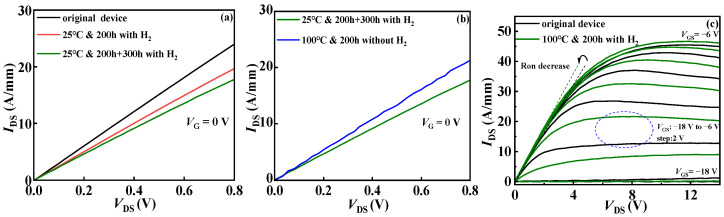
Output characteristic curves of AlGaN/GaN HEMT. (**a**) Hydrogen treatment at room temperature; (**b**) high-temperature treatment after hydrogen effect in experiment I; (**c**) high-temperature hydrogen treatment in experiment II.

It can be seen from Figure 3a that the saturation leakage current of the device decreases significantly with the increase in hydrogen treatment time at the same bias voltage at room temperature. There may be two reasons for the decrease in the output current. One is that the on-resistance becomes larger, resulting in the decrease in the output current. The other is that the output current becomes smaller due to the positive drift of threshold voltage. It is observed from Figure 3a that at room temperature, part of the current in the linear region of the output characteristic curve of the device decreases under the influence of hydrogen, indicating that the internal defects of the device are increased by hydrogen treatment at room temperature, indicating that the on-resistance Ron has changed. Therefore, it is speculated that the reason for the decrease in the output current of the device lies not only in the positive drift of the threshold voltage in the transfer characteristic curve but also in the change in Ron [12], which also indicates the corresponding decrease in the surface density of states [20,21]. It can be clearly seen from Figure 2a that the threshold voltage drifts after hydrogen treatment, which verifies the reason why the output current of the device decreases after hydrogen treatment: It is assumed that when the device is placed in the normal-temperature hydrogen environment, some new interface traps and border traps are generated at the internal SiN_x_/AlGaN interface and the AlGaN/GaN buffer interface. These new interface/border traps increase the capture of hot electrons and then suppress the output characteristics of the device. Moreover, by comparing the output characteristics of 200 h and 500 h in Figure 3a, it can be seen that the degradation degree of saturation leakage current increases with the increase in interface trap concentration. It is proved that the presence of the hydrogen in the atmosphere at room temperature affects the performance of the device to a certain extent. Figure 3b,c explore the effect of the temperature of hydrogen treatment on devices. The experimental results show that high-temperature treatment increases the saturation leakage current of the device and inhibits the current collapse of the device.

**Figure 4:**
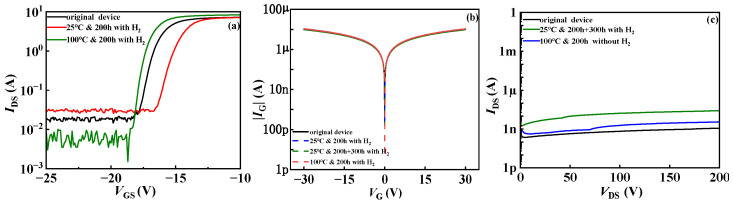
(**a**) The off-state leakage current; (**b**) the gate leakage current; (**c**) the drain leakage current of the AlGaN/GaN HEMT in experiment I.

Figure 4b is the graph of the leakage current from the gate to the source of the device. It can be seen from the data that the two variables, temperature and hydrogen, have no significant effect on the leakage current from the gate to the source. Figure 4c shows the graph of the drain to the gate leakage current of the device. The drain leakage current of the device after normal-temperature hydrogen treatment is about 10 nA, and the leakage current after high-temperature treatment is about 1 nA, which is lower than that of the normal-temperature hydrogen effect but still larger than that of the original device, considering that the high temperature time is insufficient. Figure 4a shows the transfer curves of the hydrogen effect of the device under different temperature conditions, and the ordinate is taken as the logarithmic coordinate. The off-state gate leakage current of the device was investigated under different conditions, and it was found that hydrogen treatment at room temperature increases the off-state gate leakage current of the device. However, with the treatment of high-temperature hydrogen, the gate leakage current in the off state of the device shows a downward trend, and the difference is nearly an order of magnitude. However, the saturated drain-source current increases slightly, indicating an increase in 2DEG mobility. This is relative to the previous experimental phenomenon: Under the normal-temperature hydrogen effect, due to the increase in interface traps/border traps in the SiN_x_ dielectric layer under the gate, these interface traps/border traps cause the movement of electrons inside the material to become delayed or blocked (it takes time for the trap to release electrons), and then the gate leakage current increases. After the hydrogen effect at high temperature, since the interface trap/border trap is reduced by H+ passivation, fewer electrons are trapped by the interface/border trap, so the saturation leakage current is increased.

### 3.2. The Influence of Hydrogen Effect on Interface Traps in GaN Devices

**Figure 5:**
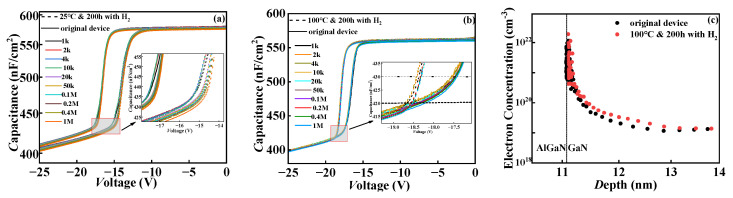
C-V characteristic curves of (**a**) hydrogen treatment at 25 °C in experiment I; (**b**) hydrogen treatment at 100 °C in experiment II. The inset shows a dispersion change; (**c**) the carrier concentration is distributed with depth in experiment II.

The C-V characteristic curves show the effect of hydrogen on the channel carrier concentration, and the test frequency was set at 1 kHz–1 MHz. To avoid positive pressure causing positive threshold drift, we only collected C-V data in the rising region of the first stage. As shown in Figure 5a, the flat band voltage of the device is positively shifted by hydrogen treatment at 25 °C. This indicates the existence of acceptor-type traps (acting as negative charge centers) at the SiN_x_/AlGaN interface/border after hydrogen treatment at room temperature, resulting in a positive shift in the flat band voltage [22,23]. The flat band voltage of the device is negative drift at 100 °C. When the gate bias voltage increases above the threshold voltage, the capacitance remains unchanged due to the formation of the 2DEG channel [20,24]. Taking the capacitance at the same frequency in the accumulation area, it can be seen that the *V*_GS_ shifts to the right after normal-temperature hydrogen treatment and to the left after high-temperature hydrogen treatment, corresponding to the right and left shifts of the threshold voltage in Figure 2a,c. The distribution curve of charge carrier concentration with depth can be extracted by using formulas as shown in follows through the C-V curve [25]:

$$\begin{array}{cc} x & =\frac{A\varepsilon_{0}\varepsilon }{C}\\ N_{t} & =-\frac{2}{q\varepsilon_{0}\varepsilon A^{2}}\times \frac{1}{\frac{dC^{-2}}{\;dV}} \end{array}$$

where *q* is the amount of electronic charge, *ε*_0_ is the vacuum dielectric constant, *ε* is the dielectric constant of AlGaN, *A* is the area of the MIS diode, and *N_t_* is the concentration of the carrier. As shown in the figure below, high-temperature hydrogen treatment increases the carrier concentration at the AlGaN/GaN heterojunction interface. It can be explained that part of the interface trap is passivated by H ions in high-temperature hydrogen, which reduces the interface trap’s capture of channel electrons and increases the carrier concentration. As a result, the saturation current of the device increases. This is consistent with the experimental results in Figure 3b of the revised manuscript.

Previous studies have demonstrated that the variation in dispersion can reflect the density of interface traps in gate media [26,27]. By comparing the illustrations in Figure 5a,b, it can be seen that the device dispersion increases after normal-temperature hydrogen treatment, which verifies the previous conjecture about the increase in interface defects in SiN_x_/AlGaN and AlGaN/GaN buffer layers and also explains the reason for the slow charge trapping in the dielectric resulting in C-V hysteresis. The hydrogen effect mainly increases the interfacial quasi-acceptor defects at 25 °C. The reason for the negative offset of the flat band voltage caused by hydrogen treatment in Figure 5b is that a large number of negative interface traps are passivated by H+ at 100 °C.

### 3.3. The Influence of Hydrogen Effect on Border Traps in GaN Devices

**Figure 6:**
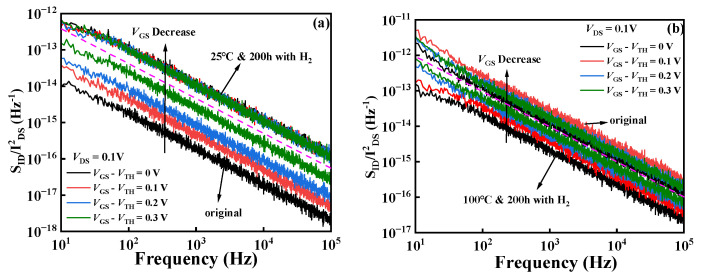
The power spectral density of the leakage current noise of the AlGaN/GaN HEMT. (**a**) Hydrogen treatment in experiment I; (**b**) hydrogen treatment in experiment II.

In order to analyze the distribution trend of the border well along the gate direction, the low-frequency noise of the devices in experiment I and II was measured. As shown in Figure 6, when *V*_DS_ = 0.1 V, the order of magnitude of *S*_id_/*I*^2^_ds_ inside the device increases with the increase in the overdrive voltage (*V*_GS_−*V*_TH_). When the frequency is 10 Hz and the overdrive voltage is 0–0.3 V, Figure 6a shows that the order of magnitude of *S*_id_/*I*^2^_ds_ of the device increases from 10^−13^~10^−14^ to 10^−12^~10^−13^ in experiment I. The increase in the noise power spectral density of the device indicates the increase in the internal border defects of the device. Combined with the above current characteristics such as the positive shift in the threshold voltage and the decrease in the saturation leakage current of the device after the normal-temperature hydrogen treatment, it is speculated that some new border traps are generated at the SiN_x_/AlGaN dielectric layer inside the device under the normal-temperature hydrogen conditions. Figure 6b shows that the *S*_id_/*I*^2^_ds_ order of magnitude decreases from 10^−11^~10^−12^ to 10^−12^~10^−13^ in experiment II. The reduction in the noise power spectral density of the device indicates the reduction in the total border defect trap inside the device, which shows better noise characteristics. It is speculated that H_2_ is decomposed into hydrogen ions through the catalysis of metal in the high-temperature environment and enters the inside of the device. It combines with the traps at the border of the SiN_x_ dielectric layer, the AlGaN barrier layer, and the GaN buffer layer; passivates the hanging bond and the interface state; and then reduces the traps inside the device.

The defect density of the border trap is extracted according to the tunneling theoretical model [11,28,29,30]. The power spectral density *S_vbf_* of flat band voltage noise and the drain current Ids have the following equation relationship:

$$\frac{S_{id}}{I_{\mathrm{d}s}^{2}}={\left(\frac{g_{m}}{I_{\mathrm{d}s}}\right)}^{2}\cdot s_{vbf}$$

$$s_{vbf}=\frac{q^{2}kT\lambda N_{it}}{wLfC_{b}^{2}}$$

The *S_vbf_* was adjusted as the input spectral noise density to achieve a good fit to the data, and the transconductance *g_m_* and operating current *I_d__s_* of the device were extracted from the measured data. *q* is the electron charge, *k* is Boltzmann’s constant, *λ* is the tunneling attenuation distance during carrier injection, which is generally 0.5 nm, *N_it_* is the density of defect states, *WL* is the gate channel area, *f* is the fixed test frequency, and *C_b_* is the series capacitance of the AlGaN barrier and SiN_x_ medium.

**Figure 7:**
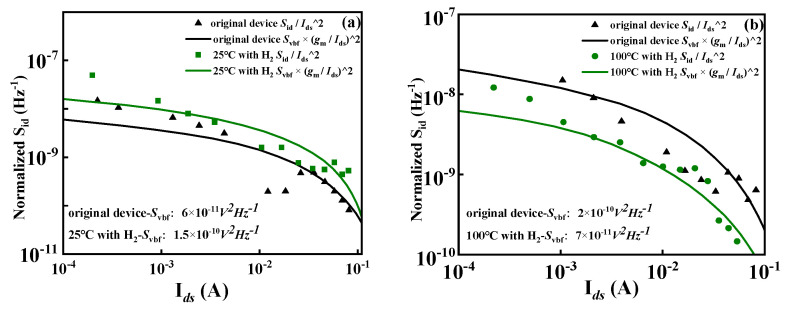
The relationship between the normalized noise power spectral density and the leakage current of the AlGaN/GaN HEMT device in (**a**) experiment I; (**b**) experiment II.

It can be seen from the fitting curve in Figure 7 that the relationship between the normalized noise power spectrum *S_id_*/*I*^2^*_ds_* and the leakage current has a good correlation with (*g_m_*/*I_ds_*)^2^ × *S_vbf_*. According to the carrier number fluctuation theory, the flat band voltage noise *S_vbf_* of the device after hydrogen treatment at room temperature increases from 6 × 10^−11^ V^2^Hz^−1^ to 1.5 × 10^−10^ V^2^Hz^−1^. The flat band voltage noise *S_vbf_* of the device after high-temperature hydrogen treatment decreases from 2 × 10^−10^ V^2^Hz^−1^ to 7 × 10^−11^ V^2^Hz^−1^. The border defect density of the device under two experimental conditions was calculated. The state density of the border defect increased from 1.1843 × 10^22^ cm^−3^eV^−1^ to 3.0337 × 10^22^ cm^−3^eV^−1^ after hydrogen treatment at room temperature. The density of border defect states decreases from 3.9477 × 10^22^ cm^−3^eV^−1^ to 1.1409 × 10^22^ cm^−3^eV^−1^ after high-temperature hydrogen treatment. Experiments with low-frequency noise further verify the conjectures of the previous mechanism: Hydrogen produces some new border traps in the SiN_x_/AlGaN dielectric layer of AlGaN/GaN HEMT under normal-temperature experimental conditions. However, in the high-temperature hydrogen effect experiment, the trap located at the border of the SiN_x_/AlGaN dielectric layer is passivated by H+, thereby reducing the trap trapping effect.

### 3.4. Interface/Border Trap Trapping Mechanism under Different Temperature Hydrogen Effects

**Figure 8:**
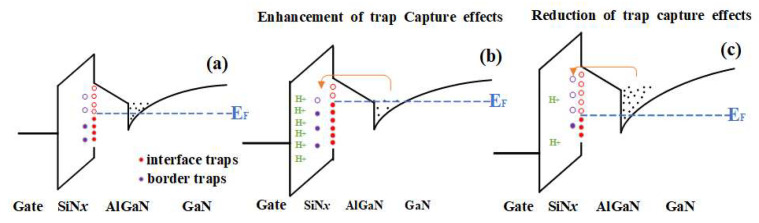
(**a**) Original device; (**b**) room temperature; (**c**) high temperature; hydrogen treatment distribution energy level diagram of interface trap/border trap.

We observe a high correlation between C-V curves and low-frequency noise after hydrogen experiments with different temperature conditions. It is fully proved that the drift of *V*_TH_ caused by hydrogen is not only affected by the interface trap but also by the border trap inside the SiN_x_ dielectric layer. In the hydrogen experiment at room temperature, because the atomic radius of H atom is smaller than that of Ga, N, and Si, some hydrogen atoms exist in the form of interstitial impurities. In the SiN_x_ dielectric layer, due to the strong electronegativity of N, the ionization energy and affinity energy are larger than H, and the hydrogen atom loses the outermost electron to become H+. As shown in Figure 8, E_F_ represents the position of the Fermi level, which is negatively charged when the defect is below the Fermi level and electrically neutral when it is above the Fermi level [12,20]. In the high-temperature hydrogen effect experiment shown in Figure 8c, due to the high thermal activity of H+, some interface traps located in SiN_x_/AlGaN and border traps located inside the SiN_x_ medium layer are passivated by H+, reducing the number of interface traps of SiN_x_/AlGaN and border traps inside the SiN_x_ medium layer. The ability of interface/border traps to capture 2DEG in conductive channels is reduced. On the other hand, the depletion of negatively charged defects located at the E_F_ level leads to a net positive charge, which exacerbates the negative drift of the threshold voltage [12]. The amount of H+ was intentionally removed when drawing Figure 8c to highlight the role of H+, which is filled in large amounts in the SiN_x_ dielectric layer in the actual hydrogen effect device.

## 4. Conclusions

In summary, this experiment investigated the effect of hydrogen treatment at different temperatures on the interface traps and border traps of depletion mode GaN HEMT devices with SiN_x_ gate dielectrics. It is also found that the device threshold voltage *V*_TH_ offset has a strong correlation with the interface/border trap concentration characterized by C-V characteristics and low-frequency noise. Therefore, we conclude that the drift of the device threshold voltage *V*_TH_ after hydrogen experiments at different temperatures is not only caused by the interface trap but also by the border trap of the SiN_x_ dielectric layer below the gate, which is proved by two experiments. It is known that hydrogen can passivate interface traps and border traps at high temperature, thereby reducing the trapping effect and reducing the density of border traps in the device. However, the relationship between the hydrogen effect of GaN devices with different structures and different working modes and the temperature of the experimental conditions has not been explored. Therefore, it is necessary to further study the long-term effects of hydrogen effect and high-temperature stress on devices with different operating modes.

## Data Availability

Data are contained within the article.
